# Serial Knockout Isolation Technique (SKIT): targeted isolation of multi-antagonist bacteria

**DOI:** 10.1016/j.mex.2026.104082

**Published:** 2026-07-31

**Authors:** Hemant Singh Maheshwari, Aakash Gour, Laxman Singh Rajput, Sanjeev Kumar, Aalok Shiv, Deepika Parmar, Palak Solanki, Jeberlin Prabina Bright, Rakesh Kumar Singh, Mahaveer Prasad Sharma

**Affiliations:** a ICAR-National Soybean Research Institute, Indore, 452001, India; b University of Groningen, Groningen, 9747AG, The Netherlands; c Rajmata Vijayaraje Scindia Krishi Vishwavidyalaya, Gwalior, 474002, India; d ICAR-Central Arid Zone Research Institute, Jodhpur, 342003, India; e Tamilnadu Agricultural University, Coimbatore, 641003, India

**Keywords:** Serial Knockout Isolation Technique (SKIT), Serial Enrichment Incubation Technique (SEIT), Triple sugar iron test (TSI), Siderophore-producing bacteria (SPB), Siderophore, Hydrocyanic acid (HCN), Antagonistic bacteria, Dual assay

## Abstract

Traditional approaches to isolating antagonistic bacteria are non-targeted, yield lower recovery of potential multi-antagonists, and require substantial time, human resources, and lab resources. We advocate isolating siderophore-producing bacteria (SPB) or hydrolytic enzyme-producing bacteria (cellulase, protease, or chitinase) using the Serial Enrichment Incubation Technique (SEIT). Further, these recovered bacteria may be tested using the Triple Sugar Iron (TSI) test, which can easily identify Enterobacteriaceae and exclude probable human pathogens. Then, the Hydrocyanic (HCN) production assay may be performed, and prioritize both siderophore- and HCN-positive bacterial isolates in a dual assay against the predominant target phytopathogens first, then others. Here, non-siderophore and enterobacteriaceae members are serially eliminated or knocked out using the Serial Knockout Isolation Technique (SKIT). SKIT is proposed to reduce workflow time for isolating multi-antagonist bacteria, thereby saving time, labor, and lab resources.

## Highlights

• SKIT combines SEIT, TSI, and HCN assay, followed by a dual assay for isolation of multiple antagonistic bacteria.
• SKIT provides a focused approach and reduces the likelihood of recovering Enterobacteriaceae and workflow time compared to traditional screening.
• SKIT involves serially knocking out the recovered bacteria by screening against the predominant target phytopathogens in a decreasing order.
• Recovery of isolating potentially multi-antagonist through SKIT is higher than traditional screening.

## Graphical abstract

**Figure:**
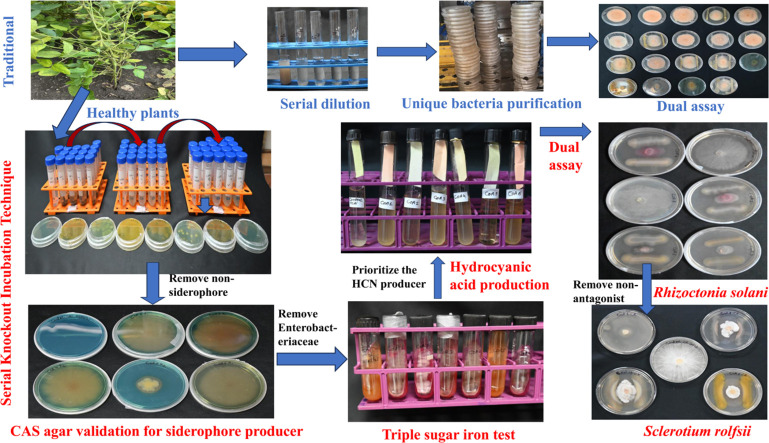

## Specifications table

**Table:** 

**Subject area**	Agricultural and Biological Sciences
**More specific subject area**	Plant pathology
**Name of your method**	Serial Knockout Isolation Technique (SKIT): targeted isolation of multi-antagonist bacteria
**Name and reference of the original method**	NA
**Resource availability**	Included in the method details section

## Background

A single pathogen initially causes plant diseases; however, over time, multiple pathogens may be involved. Soybean seedlings are commonly challenged by several soil-borne pathogens during the initial stages of crop establishment, including *Rhizoctonia solani, Macrophomina phaseolina, Agroathelia rolfsii*, and *Fusarium solani*. However, over time, these pathogens infect soybean plants in complex ways, making it difficult to distinguish the disease. Further, even minor diseases are becoming major diseases due to climate change [1]. These show that, over time, due to climate change, multiple or complex diseases are more common than single diseases [2]. Therefore, to combat such complex diseases, isolating multi-antagonist bacteria is needed.

A traditional way to isolate antagonistic bacteria or microbes involves serially diluting healthy plant or soil samples, then spreading the diluted samples onto a common medium, such as nutrient agar, Luria-Bertani agar, or tryptic soy agar. Using traditional approaches, more than 4 unique bacteria per sample are isolated, which is difficult to handle and to conduct screening tests such as biochemical, plant growth promotion, and antagonistic tests, and then finally identify 1-2 potential ones [[3], [4], [5]]. In these approaches, the risk of human pathogens is also very high [6,7]. Fourteen bacteria were isolated from mungbean rhizosphere samples based on morphological traits and then screened for various traits. It was found that *Bacillus amyloliquefaciens* D5 showed antagonism against leaf spot disease and had potential for plant growth promotion [4]. Another study showed that antagonistic bacteria isolated from 40 rhizospheric samples recovered 118 strains and, after rigorous screening, found that *B. amyloliquefaciens* P10-7 showed antagonism against *Botrytis cinerea* [3]. Twenty yeast isolates from seeds, and among these, two *Issatchenkia terricola* GRY4 and *Pichia Kudriavzevii* POY5 showed many plant growth promotion tests, including siderophore and control *R. solani* in black gram [8]. Two forty-seven actinomycetes isolates were isolated from the rhizosphere of forest plants, among which *Streptomyces olivoreticuli* was identified as a biocontrol agent against *R. solani* [9]. Similarly, isolated *Streptomyces cellulosae* AT6-60 as a plant growth promoter and biocontrol agent from tomato rhizosphere samples, following screening of 208 isolates for various plant growth-promoting traits and biocontrol against soil-borne pathogens [5].

Among the above recent studies on the isolation of bacteria [3,4], yeast [8], and actinomycetes [5,9], the final screened antagonist shows siderophore-producing traits. Additionally, a common observation in our lab during isolation of antagonist bacteria is that siderophore-producing bacteria exhibit antagonistic activity. Further, among all siderophore-producing bacteria, hydrocyanic acid assay bacteria showed the greatest antagonistic activity. Therefore, we hypothesized that if any protocol selectively allows the growth of siderophore-producing bacteria from a sample, the recovered bacteria may be further tested in the HCN assay, which would most likely yield potential antagonists. Additionally, the presence of Enterobacteriaceae in recovered isolates can be differentiated using the triple sugar iron test, which excludes probable Enterobacteriaceae members and focuses on the remaining bacteria.

## Method validation

The mechanism of antagonistic bacteria is mediated through induced systemic resistance and the production of lytic enzymes, lipopeptides, and siderophores [10]. Taking siderophore, cellulase, or chitinase as a key criterion, screens only a few desirable bacteria from the sample. Aiming for these key traits as criteria allows likely non-antagonistic bacteria to be eliminated during the isolation procedure, compared to the conventional isolation technique. Siderophores are iron chelators that provide iron under iron-deficient conditions, and siderophore-producing bacteria (SPB) are involved in the suppression of phytopathogens by depriving them of iron nutrition. Common SPB bacterial genera reported for controlling phytopathogens include *Bacillus, Pseudomonas, Azotobacter, Rhizobium, Alcaligenes,* and *Acinetobacter* [11].

## Serial Knockout Isolation Technique (SKIT) approaches

The current SKIT approach advances our traditional isolation technique. Similar to the knockout stages of football, where every match is a must-win for the winner, the SKIT technique emphasizes isolating bacteria using the SEIT [12]. In these techniques, fewer potential bacteria are isolated than in conventional techniques. Here, we advocate isolating bacteria from rhizospheric samples (1 g) from disease-free plants, or from surface-sterilized crushed plant tissue (1 ml) for isolating endospheric microorganisms by incubating in iron-free succinate media [12]. Recovered cultures are then subjected to the TSI test, which allows elimination of Enterobacteriaceae, a group that includes some human-pathogenic members, based on gas production or color [13]. Further, an HCN production test was carried out, and positive ones were further tested against phytopathogens in a dual assay in priority. For example, in soybeans, *R. solani, M. phaseolina, F. solani,* and *A. rolfsii*, in decreasing order, in a sequence [1]. Then only those bacteria that show antagonistic action should be chosen for the next pathogen, and so on. Here, one bacterium that shows antagonism against one pathogen but not against another will be eliminated. The final winners in this context would be a multi-antagonist bacterium. This process of serial elimination is termed the serial knockout isolation technique (SKIT). In this improved approach, we may also use cellulose or chitin as the key substrate and enrich bacteria present in the sample that can catabolize it, which is a key constituent of any fungal cell wall. Since biocontrol bacteria release cellulase or chitinase enzymes, which help degrade the pathogenic fungus's cell wall [[14], [15], [16]].

## Chemicals or equipment required

Agar-Agar, Type I (Himedia Laboratory, India; GRM 666)

Potato dextrose agar (Himedia Laboratory, India; SKU: SM096)

Nutrient broth (Himedia Laboratory, India; SKU: M002)

Sodium chloride (Himedia Laboratory, India; TCO46)

Sodium hydroxide pellets for analysis EMSURE® 1310-73-2 Merck

Hydrochloric acid 37%, for analysis, EMSURE® ACS, ISO, Reag. Ph Eur (7647-01-0)

K_2_HPO_4_ (Himedia Laboratory, India; SKU: GRM 1045)

KH_2_PO_4_ (Himedia Laboratory, India; SKU: PCT0009)

Cetrimonium bromide (CTAB) (Himedia Laboratory, India; SKU: MB101)

(NH_4_)_2_SO_4_ for analysis EMSURE® ACS, ISO, Reag. Ph Eur 7783-20-2 Merck

MgSO_4_.7H_2_O for analysis EMSURE® ACS, Reag. Ph Eur 10034-99-8 Merck

Succinate (Himedia Laboratory, India; SKU: PCT0504)

Iron (III) chloride anhydrous for synthesis 7705-08-0 Merck

Chromeazurol S Dye content 45 % 199532 Sigma-Aldrich

Peptone granulated (Himedia Laboratory, India; SKU: RMG7709)

Yeast extract powder (Himedia Laboratory, India; SKU: RMO27)

Nystatin, Cell culture tested (Himedia Laboratory, India; TC032)

Dimethyl sulfoxide ACS, 99.9 % (28,580 SRL)

Glycine (Himedia Laboratory, India; SKU: MB013)

Sodium Carbonate anhydrous (Himedia Laboratory, India; GRM254)

Picric acid (Himedia Laboratory, India; RM 1876)

Beef extract, powder type 1 (Himedia Laboratory, India SKU: RM669)

Peptone, Bacteriological (Himedia Laboratory, India SKU: RM001)

Glucose (Himedia Laboratory, India SKU: GRM016)

Lactose (Himedia Laboratory, India SKU: GRM017)

Sucrose (Himedia Laboratory, India SKU: GRM601)

Sodium thiosulfate anhydrous (Himedia Laboratory, India SKU: GRM1420)

Ferrous sulfate (Himedia Laboratory, India SKU: GRM4376)

Ethanol (70 %)

Laminar air flow

Autoclave

Analytical balance 220 g/0.1 mg capacity

Micropipette (100–1000 µl) single-channel capacity

BOD incubator shaker pH meter.

Eppendorf tubes (50 ml and 15 ml)

Glass test tubes with black screw caps (30 ml capacity)

Inoculating loops and needles

Erlenmeyer culture flask with flat bottom (250 mL, 500 mL, and 1000 mL) capacity

Cork borer

Cell spreader

Petri dishes (90 mm × 15 mm)

Bacteriological filter 0.22 µm

Whatman paper no. 1

## Preparation of media and reagents

(1) Iron-free succinate media preparation: Composition (g L^-1^) K_2_HPO_4_-6.00, KH_2_PO_4_-3.00, (NH_4_)_2_SO_4_-1.00, MgSO_4_.7H_2_0-0.20 and succinate - 4.00 were weighed and mixed in 980 ml of distilled water. The pH was maintained at 6.80 with 1 M NaOH and 1 M HCl, and the final volume was adjusted to 1000 ml. Then 10 ml of media was dispensed into a 50 ml Eppendorf tube and autoclaved at 121°C for 20 min [17].
(2) Chromo Azurol sulfonate (CAS) agar plate preparation: Composition (solution A: 60.50 mg Chromoazurol sulfonate mixed in 50 mL of deionized water, Solution B: dissolve 2.70 mg of ferric chloride in 10 mM HCl, and Solution C: 72.90 mg of CTAB was dissolved in 40 mL of distilled water. Now, solution A was mixed with solution B while stirring, and then the blue color appeared. Finally, solutions A and B were mixed with solution C to obtain a dark blue color. This solution was autoclaved separately.

Further, 900 ml of Nutrient broth (composition: Peptone-5.00, Yeast extract-3.00, Sodium chloride-5.00, and Agar-agar-18.00 g) was prepared and autoclaved separately [18]. After autoclaving 100 ml of CAS dye mixed with 900 ml of nutrient agar, the mixture is cooled to approximately 45-50°C, then poured into Petri dishes. The conversion of blue plates to an orange halo was interpreted as positive for siderophore production. Siderophore index was calculated using the following equation [19].

SI=Halozonediameter/Colonydiamter

Where SI represents the siderophore index.

A ratio greater than 1.0 is considered a significant siderophore producer [19]. Here, 5 µL of bacterial culture was spot-inoculated onto a CAS agar plate in triplicate, incubated at 28°C, and the halo zone and colony diameter were recorded after four days.

(3) Nystatin antibiotic preparation: A stock solution (50 mg mL^-1^) was prepared by dissolving 500 mg Nystatin (PCT1112, Himedia Laboratory, India) into dimethylsulfoxide (DMSO) and then filter sterilized with a bacteriological filter with a size of 0.22 µm and placed into a sterile 15 ml Eppendorf tube and wrapped in aluminium and stored at 4°C in a refrigerator. It was added to iron-free succinate media, CAS agar plate, and Nutrient broth & agar at 50 µg mL^-1^ to check for fungal contamination.
4) Potato dextrose agar (PDA), Nutrient agar (NA), and nutrient broth (NB) preparation:

For PDA preparation, 39 g were dissolved in 950 ml of water, heated in a microwave until fully dissolved, the pH was maintained at 5.6 ± 0.2, and the volume was made up to 1000 ml. Similarly, for nutrient agar preparation, 45 g was taken, dissolved in 950 ml, heated to dissolve all contents, the pH was maintained at 7.6 ± 0.2, and the final volume was made to 1000 ml. Nutrient broth was prepared by dissolving 13 g in 950 ml of distilled water, heating to ensure complete dissolution, and maintaining the pH at 7.4 ± 0.2. The final volume was made to 1000 ml by mixing additional distilled water. These two were then autoclaved at 121°C for 20 min. It was allowed to cool to approximately 45-50°C at room temperature, then poured into sterile Petri dishes in a laminar airflow.

(5) Triple sugar iron (TSI) media

Composition of TSI medium (gL^-1^): Beef extract-3.00; yeast extract-3.00; Peptone-20.00; Glucose-1.00, Lactose-10.00, Sucrose-10.00, Ferrous sulfate-0.20 g, NaCl-5.00, sodium thiosulfate 0.30, phenol red-0.024 and agar- 18.00). Here, all its constituents except agar-agar were weighed, and 950 ml of water was added. The pH was maintained at 7.30, and then agar-agar was added. The final volume was maintained at 1000 mL. Then the 10 ml medium was dispensed into a 30 ml glass tube with black screw caps and was autoclaved at 121°C for 20 min. After autoclaving, slants were prepared by placing the glass tubes at an angle of 45°. Bacterial cultures were stab-inoculated into a butt, streaked on the surface of a slant, and incubated at 28 ± 2°C for 4 days. Observations were made related to the discoloration of the slant and butt, which turned yellow or red, and gas and H_2_S production was observed (Table 1). Here, bacterial culture producing gas, H_2_S, and acid/acid may be excluded as a probable member of Enterobacteriaceae [13].

(6) Hydrocyanic acid production:

**Table 1:** Triple sugar iron interpretation [13].

Sr. no	Slant/Butt	Description	Possible members	SKIT Knockout
1.	Yellow/Yellow	Bacteria are fermenting glucose, lactose, and sucrose.	*Enterobacter aerogenes, E. cloacae, Escherichia coli, Klebsiella*	Reject
2	Yellow/Yellow + Black precipitate	All three sugars are fermented, and bacteria produce hydrogen sulfide.	*Citrobacter spp., Proteus spp.*	Reject
3.	Red/Red or Red/No change	All three sugars are not metabolized. Non-fermenting bacteria.	*Pseudomonas* spp. and *Acinetobacter* spp.	Retained
4.	Red/Yellow + Black precipitate	Glucose fermenter and H_2_S producer.	*Citrobacter, Edwardsiella, Proteus* spp., and *Salmonella* spp.	Reject
5	Red/ No change + Black Precipitate	A glucose non-fermenter that produces hydrogen sulfide.	*Shewenella*	Reject
6.	Gas Production	Splitting of agar or sometimes agar is raised above its original position.	*E. coli, Enterobacter* spp., and *Klebsiella* spp.	Reject

The HCN assay was performed with slight modifications [20]. Briefly, nutrient broth was prepared as discussed above and mixed with glycine (0.44 %). Then dispense 10 mL of broth into a 30 mL glass tube with screw caps and autoclave at 121°C for 20 min. After autoclaving, the media were cooled, and 10 µl of the recovered bacterial suspension was inoculated. Another 100 ml solution of 2 % Na_2_CO_3_ was prepared and autoclaved separately. After autoclaving, the solution was cooled, 0.5% picric acid was added, and the solution was filter-sterilized. Then, sterilized Whatman paper no. 1 was dipped into the solution and hung on the inoculated media, and the screw caps were closed. These tubes are incubated for 4 days at 28 ± 2°C. Discoloration of the control from yellow to orange or reddish- brown was considered positive.

## Sample collection

Six rhizosphere samples were collected from healthy plants and processed further using both traditional and SKIT approaches to isolate multi-antagonist bacteria.

**Approaches for isolation of multi-antagonist bacteria**

(1) Traditional approach

1 g of rhizosphere samples from healthy plants was collected and stored in a 4°C mini water cooler before being taken to the laboratory. Then, 1 g samples were transferred to 9 ml sterile distilled water and diluted serially 10^-1^, 10^-2^, 10^-3^, 10^-4,^ and 10^-5^. From the final tubes, 10^-5^, 100 µL was uniformly spread onto nutrient agar and incubated at 28°C for 48 h; a unique colony appearing on the media was further purified in nutrient agar Petri dishes. Then, purified bacterial cultures were grown in nutrient broth at 28°C for 2 days and stored at 4°C. These freshly grown cultures were used for further research.

(2) SKIT approach

1 g of rhizosphere soil was taken and placed directly into 10 ml of iron-free succinate medium amended with nystatin (50 µg mL^-1^) in a 50 ml Eppendorf tube. These tubes were incubated for 5 days at 28°C with 120 revolutions per minute (rpm). Then 100 µl of suspension was transferred to the new media and incubated as described above. This step was done twice. From the final incubated tubes, 1 mL was taken, serially diluted to 10^-5^, and 100 µL of the suspension was spread uniformly onto the CAS agar plates. These plates were kept in a BOD incubator at 28°C, and then unique colonies were purified into CAS plates. These unique colonies were grown, inoculated into nutrient broth, incubated at 28°C for 2 days, and then stored at 4°C for further research [12].

**Fig. 1:**
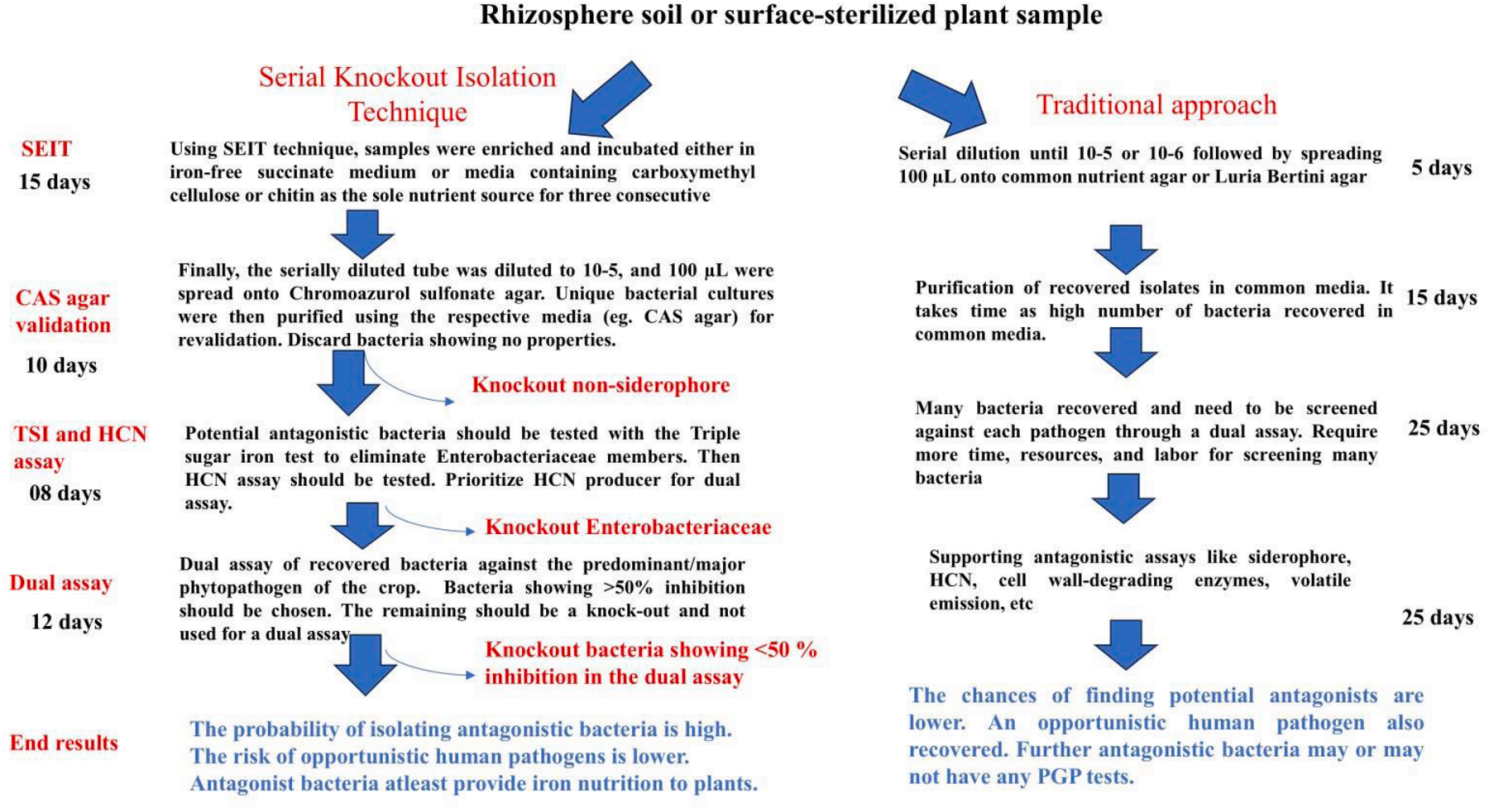
Step-wise protocol comparison between the Serial Knockout Isolation Technique and the traditional approaches.

All recovered bacteria were tested for the TSI test [13], and probable Enterobacteriaceae were eliminated. Here, yellow/yellow, yellow + black, red/red + black, and gas producer were removed. The remaining bacteria are then assessed in the HCN production assay, and prioritized HCN producers are selected for the dual assay. Since these TSI and HCN tests were not costly or resource-intensive, any researcher can easily exclude possible human pathogens and focus on the remaining one for further assay (Fig. 1).

## Dual assay of bacteria recovered through traditional and SKIT against soybean phytopathogens

The dual assay of bacteria recovered using traditional and a SKIT method was assessed against four predominant soil-borne phytopathogens of soybean: *R. solani, M. phaseolina, F. solani*, and *A. rolfsii*. Here, these four phytopathogens were collected from the Plant Pathology Lab, ICAR-NSRI, Indore. These cultures were initially grown on freshly prepared potato dextrose agar and incubated at 28°C for five days. For the dual assay, potato dextrose agar plates were prepared. Then, bacterial cultures recovered by SKIT and traditional methods were streaked in parallel on a Petri dish and incubated at 28°C for 24 h; the respective fungal discs were then taken using a sterilized 0.8 cm cork borer and placed at the center; the experiment was performed in triplicate. The Petri dishes were then wrapped in parafilm and incubated for another 4 days. Here, a control plate without bacterial culture of the respective fungi was maintained in triplicate. Phytopathogen mycelial growth inhibition by the recovered bacteria was measured relative to the control and expressed as the inhibition percentage (% I) using the following equation [21].

$$\%I=\left(1-\frac{T}{C}\right)\times 100$$

Where, I = Phytopathogen inhibition (%), C = radial growth of fungi in control (cm), T = radial growth of fungi in the presence of test bacteria (cm)

In a dual assay, we did not find any literature providing a cut-off for %I phytopathogen inhibition. Therefore, for the present investigation, we used > 50% of the bacteria to demonstrate strong antagonistic activity. Sometimes negative results appear, indicating that the test fungi's growth increases in the presence of bacteria.

## Pathogen priority list

The pathogen priority list crop- and location-specific. It varies across and within agroclimatic zones as the disease triangle shows the interaction among pathogen, host, and environment. Further, the extent of crop losses caused by the pathogens may also be a factor in determining pathogen priority lists. Consequently, pathogen priority lists should be adapted according to the regional prevalence and economic importance of target pathogens [[22], [23], [24], [25], [26]]. Additionally area of interest of researcher for targeted pathogen may varies with choice choice of the researcher (Table S1).

## Biosafety

Since bacteria are isolated from rhizosphere samples, preliminary tests such as TSI can identify Enterobacteriaceae. Such a probable human pathogen should be discarded as biohazardous waste by placing it in an autoclavable biohazard bag and autoclaving it at 121°C for 20 min. The sterilized waste should be disposed of through the designated municipal waste collection agency. During handling, wear gloves, lab protection, and eye protection.

## Statistical analysis

Percent inhibition of recovered bacterial isolates using both traditional and SKIT was analyzed using Welch's t-test and one-way Analysis of Variance (ANOVA) in IBM SPSS 31.0. Post-hoc comparisons were performed using Duncan's Multiple Range Test (DMRT) at 5 %, and results were presented as mean ± standard deviation, followed by lowercase letters indicating significant differences.

## SKIT

**Fig. 2:**
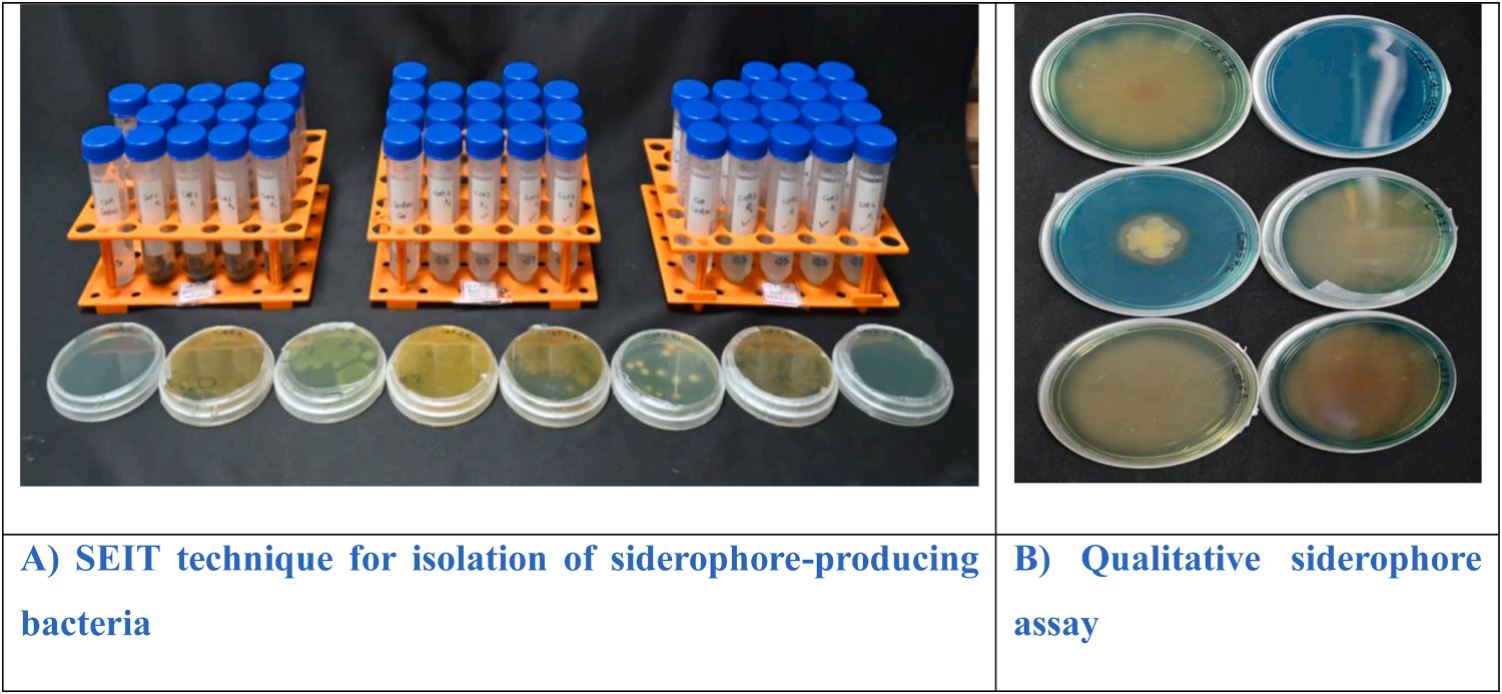
Isolation of siderophore-producing bacteria through the SEIT approach and qualitative siderophore assay.

**Fig. 3:**
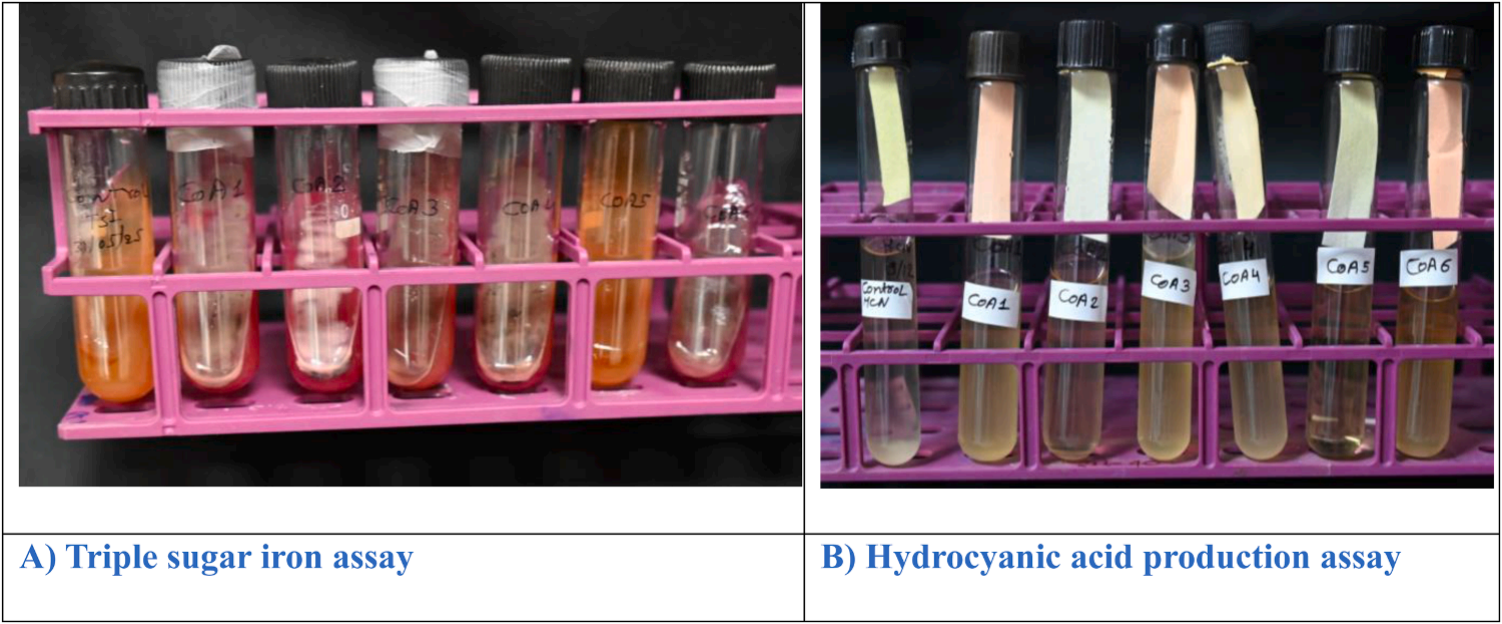
Triple sugar iron and hydrocyanic acid production assay of the SKIT recovered isolates.

In the present investigation, only 6 unique bacteria were recovered from 6 rhizospheric samples using SKIT, whereas 18 were recovered using traditional screening. The dual assay showed that among 6 bacteria recovered by the SEIT technique (Fig. 2), COA2 and COA5 may be excluded as possible enterobacteriaceae members, and further, these two were HCN negative (Fig. 3 and Table 2) and do not control *R. solani* and *M. phaseolina* phytopathogens; therefore, they were not tested further for *F. solani* and *A. rolfsii*. Further, COA2 was a non-siderophore producer.

**Table 2:** Qualitative evaluation of SKIT recovered bacterial isolates for siderophore, triple sugar iron, and HCN production.

Bacterial isolate	Siderophore production	Triple sugar iron test	HCN production assay	Decision
COA1	+	Red/Red	+	Retained
COA2	-	Red/Red	-	Rejected
COA3	+	Red/Red	+	Retained
COA4	+	Red/Red	+	Retained
COA5	+	Yellow/Yellow	-	Rejected
COA 6	+	Red/Red	+	Retained

**Fig. 4:**
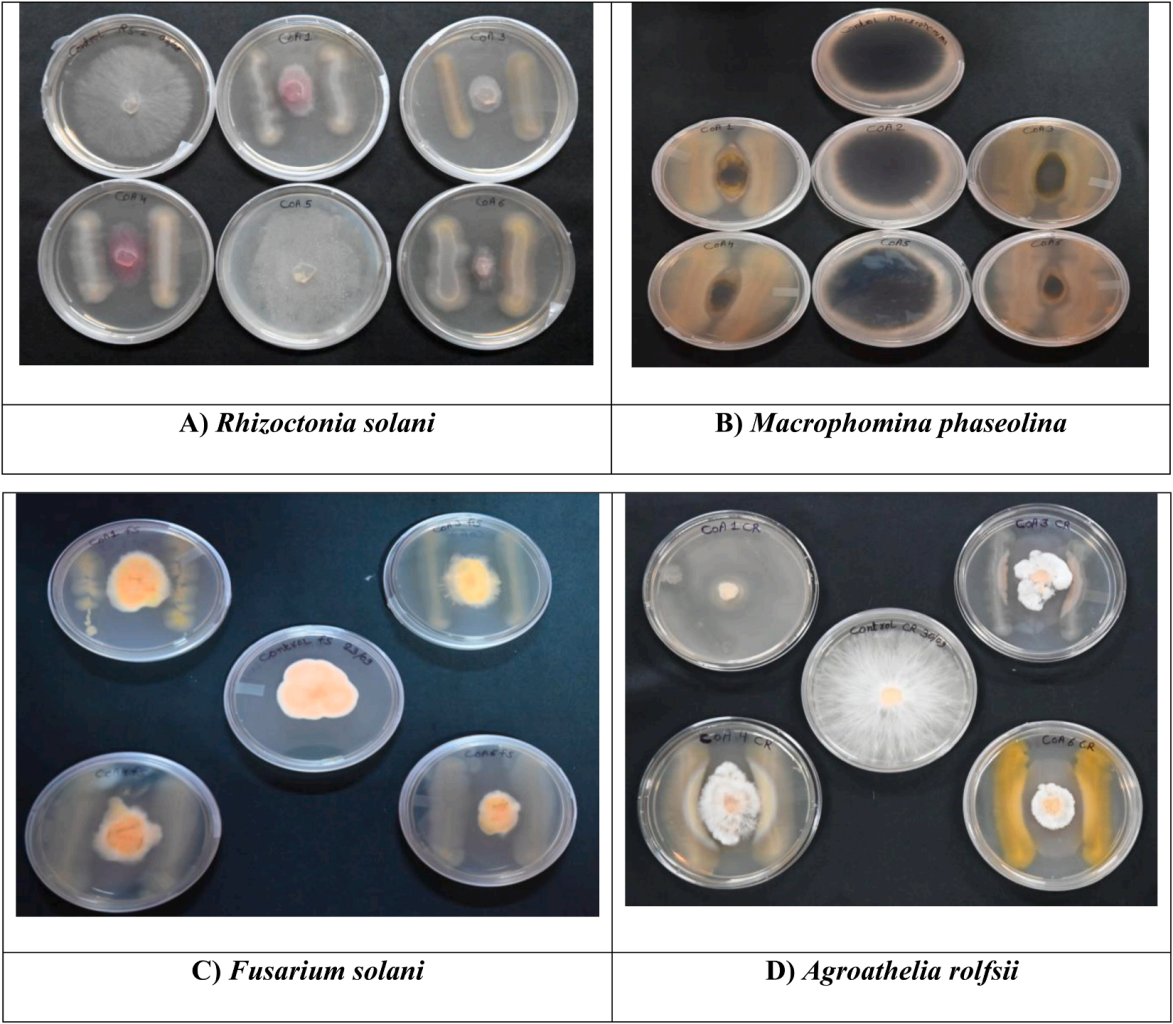
Evaluation of bacteria recovered through the SKIT approach against predominant soybean phytopathogens.

Dual assay against all four soil-borne soybean phytopathogens showed that the remaining four bacteria, namely COA1, COA3, COA4, and COA6, were multi-antagonist. This approach reduced bacterial recovery and the likelihood of becoming an opportunistic human pathogen. The further antagonistic potential of the SKIT-recovered bacteria was much higher than that of bacteria recovered through traditional methods. All the recovered bacteria through SKIT control *R. solani* equally. However, in *M. phaseolina*, COA4 followed by COA6 showed greater inhibition potential. Against *F. solani*, COA 6 followed by COA 1 showed greater inhibition. In contrast, COA1 followed by COA6 showed inhibition against *A. rolfsii* (Fig. 4 and Table 3).

**Table 3:** Percent inhibition of bacterial isolates recovered through the SKIT approach.

Culture name	*Rhizoctonia solani*	*Macrophomina phaseolina*	*Fusarium solani*	*Agroathelia rolfsii*
COA1	81.25 ± 1.25a	75.83 ± 0.72c	67.083 ± 0.72b	79.58 ± 0.72a
COA2	2.91 ± 1.44b	2.08 ± 0.72d	-	-
COA3	81.25 ±1.25a	77.08 ± 0.72bc	60 ± 1.25c	57.91 ± 0.72d
COA4	79.58±1.91a	80.83 ± 0.72a	60.42 ± 0.72c	62.5 ± 1.25c
COA5	0.00±0.00c	2.91 ± 0.72d	-	-
COA6	81.25 ± 1.25a	78.33 ± 0.72b	71.67 ± 0.72a	74.58 ± 0.72b
F value	2905.41	8776.567	121.11	393.94
P value	<0.001	<0.001	<0.001	<0.001

## Traditional approach

**Fig. 5:**
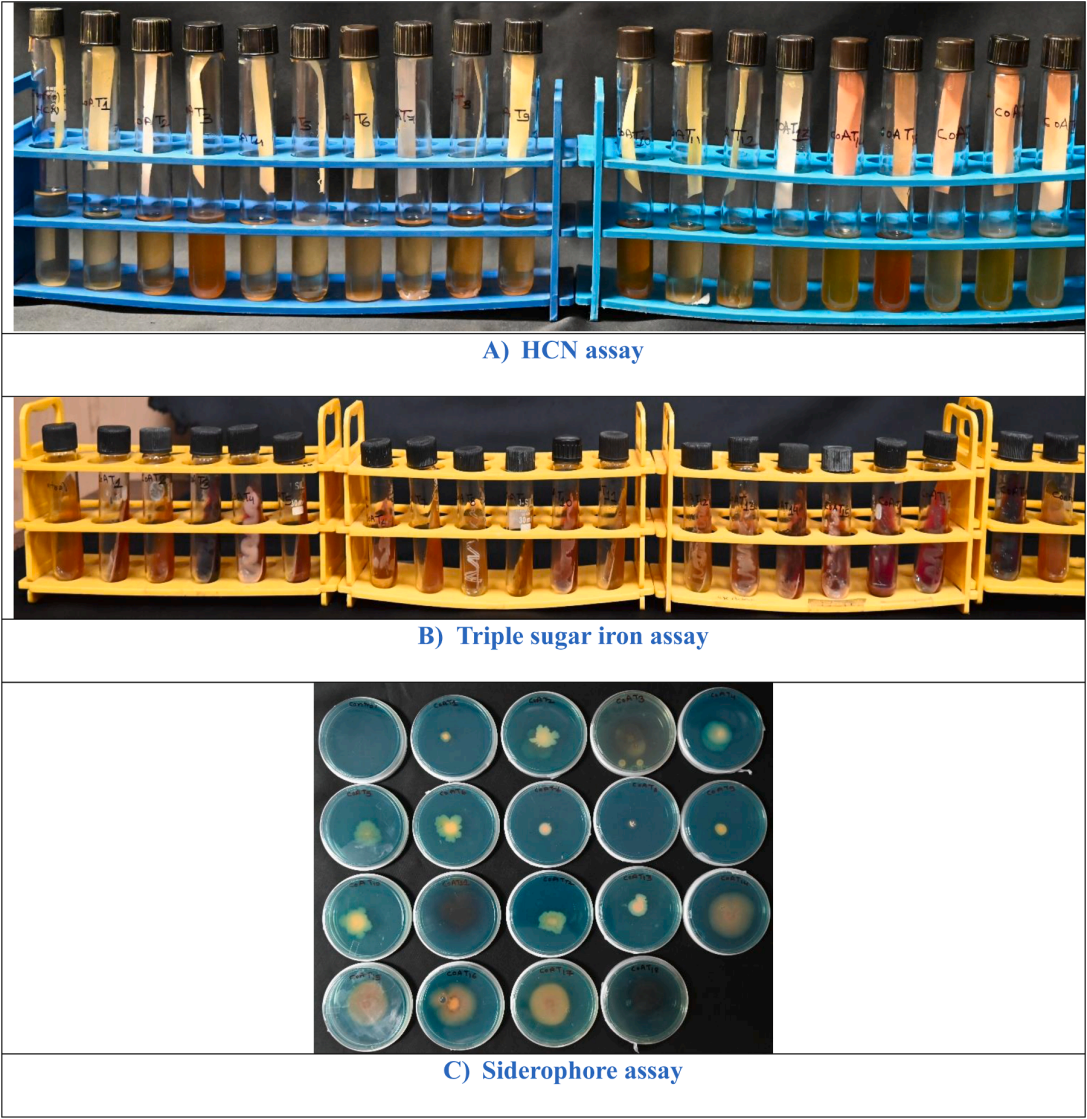
HCN production, triple sugar iron test, and qualitative siderophore assay of the bacterial isolates recovered through traditional approach .

**Fig. 6:**
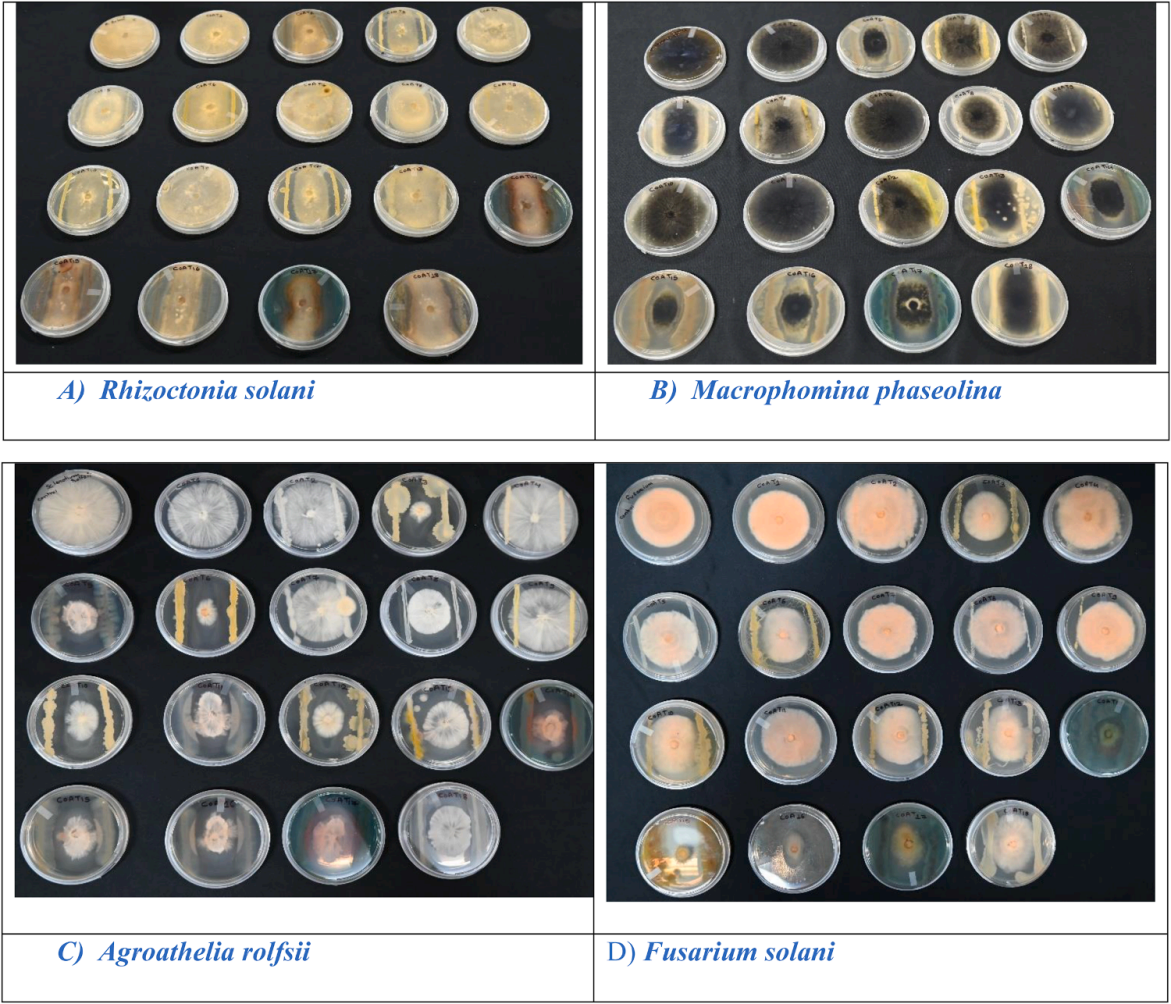
Evaluation of bacterial isolates against soybean phytopathogens isolated through a traditional approach.

In contrast, using the traditional approach, 18 bacteria were recovered, and the TSI assay showed that 10 belonged to Enterobacteriaceae. Further, 6 were siderophore- and HCN-positive (Fig. 5 and Table 4). Dual assay against all four phytopathogens showed that COAT3, COAT14, COA15, COA16, and COA17 were multi-antagonists controlling at least three phytopathogens (Table 5). However, COAT3 and COAT15 showed H_2_S and gas production, respectively, indicating a probable Enterobacteriaceae member (Table 4). Among all multi-antagonists, COA15 is a potential multi-antagonist; however, it may belong to Enterobacteriaceae as it produces gas. However, it needs to be validated by 16S DNA sequencing for identification (Tables 4 & 5), Fig. 6.

**Table 4:** Qualitative siderophore, triple sugar iron (TSI), and HCN production assay of bacterial culture recovered using the traditional approach.

Bacterial isolates	Siderophore assay	Triple sugar iron assay (Slant/Butt)	HCN assay	Enterobacteriaceae member
COAT1	-	Red/Red	-	-
COAT2	-	Yellow/Yellow	+	+
COAT3	+	Red/Red + Black H_2_S	-	+
COAT4	-	Red/Red	-	+
COAT5	-	Red/Yellow	-	-
COAT6	-	Red/Yellow	-	-
COAT7	-	Yellow/Yellow	-	+
COAT8	-	Yellow/Yellow	-	+
COAT9	-	Yellow/Yellow	-	+
COAT10	-	Red/Red	-	-
COAT11	-	Yellow/Yellow	-	+
COAT12	-	Yellow/Yellow	-	+
COAT13	-	Red/Red	-	-
COAT14	+	Red/Red	+++	-
COAT15	+	Red/Red + Gas	+	+
COAT16	+	Red/Red	++	-
COAT17	+	Red/Red	+	-
COAT18	-	Red/Red + H2S	+	+

**Table 5:** Percent inhibition of bacterial isolates recovered through traditional screening.

Culture name	*Rhizoctonia solani*	*Macrophomina phaseolina*	*Fusarium solani*	*Agroathelia rolfsii*
COAT1	2.50±2.50h	0.42±0.72g	0.00±1.61e	6.25±2.36e
COAT2	48.75±6.61de	67.92±2.60ab	-17.20±5.18f	3.99±2.83e
COAT3	51.25±1.25bc	51.67±2.60d	18.82±2.46d	71.47±1.36a
COAT4	0.42±0.72h	3.75±3.31g	-9.68±4.27f	0.81±3.59e
COAT5	21.67±5.05g	25.83±8.78e	6.45±4.27e	54.26±4.77b
COAT6	40.00±2.17f	13.33±1.44f	8.06±1.62e	64.68±7.19a
COAT7	1.25±1.25h	0.42±0.72g	3.77±0.93e	15.76±5.92d
COAT8	42.50±50ef	27.08±2.60e	3.23±1.62e	38.86±3.60c
COAT9	2.08±1.91h	0.42±0.72g	0.00±1.61e	7.16±1.57e
COAT10	55.42±4.39b-d	4.80±1.44g	4.84±5.81e	52.90±5.66b
COAT11	1.25±2.17h	2.50±3.3g	1.08±2.46e	48.37±7.56b
COAT12	55.00±3.75bcd	30.00±6.61e	4.30±3.36e	56.07±3.42b
COAT13	38.75±1.25f	18.33±1.91f	18.82±9.86d	37.05±4.15c
COAT14	56.25±4.51bc	62.92±1.91bc	52.15±9.72b	54.26±2.07b
COAT15	65.42±2.60a	72.92±1.91a	72.58±1.61a	52.45±6.23b
COAT16	58.75±4.51b	66.67±2.60b	60.22±7.27b	52.45±1.36b
COAT17	58.33±5.77bc	60.42±2.60c	40.32±7.39c	52.45±4.08b
COAT18	55.00±6.61bcd	48.75±1.25d	7.53±4.06e	49.28±2.08b
F value	114.95	205.35	81.95	73.14
P value	<.001	<.001	<.001	<.001

The current findings from the traditional approach also support the idea that siderophore- and HCN-positive isolates are more likely to be antagonistic bacteria, as potential antagonists recovered from the approach were both siderophore and HCN-positive. Further comparisons of the potential of multi-antagonist bacteria between the two approaches showed that those recovered via SKIT had greater potential than those recovered via the traditional approach for controlling phytopathogens in the same sample.

The results showed that the recovery of bacteria and the likelihood of being an Enterobactereaceae member using the traditional approach are very high (55.56 %). Bacteria isolated through SKIT have greater siderophore (83.33 %) and HCN (66.67 %) production abilities and are more likely to be multi-antagonists. Regarding time, SKIT had a 66.67% chance of recovering multi-antagonists compared to the traditional approach (27.78%) in the present investigation. Regarding time, the SKIT approach showed 35.71 % more efficiency than traditional ones (Table 6).

**Table 6:** Comparison between the Serial Knockout Isolation Technique and the traditional approach.

Approach	Number of samples taken	Number of unique bacteria recovered	Enterobacteriaceae based on TSI	Siderophore	HCN	Multi-antagonist	Time (efficiency)
SKIT	7	6	1	5	4	4	45 days
Efficiency of SKIT (%)	-	85.71 %	16.67 %	83.33 %	66.67 %	66.67 %	35.71 % efficient than Traditional
Traditional	7	18	10	5	6	5	70
Efficiency of traditional (%)	-	22.22 %	55.56 %	27.78 %	33.33 %	27.78 %	-

Results showed that bacteria isolated using the SKIT approach had a higher percent inhibition potential (49.53-349.00%) than those isolated using the traditional approach (Table 7). The SKIT approach significantly reduced variability (CV) 7.93% - 70.87% in the recovery of potential biocontrol isolates compared with the traditional approach, resulting in more consistent isolation of promising biocontrol agents. Advantages and limitations of SKIT over traditional approaches have been compared (Table 8).

**Table 7:** Differences in the multi-antagonist bacteria potential recovered using the traditional and SKIT approaches.

Approach	*Rhizoctonia solani*	*Macrophomina phaseolina*	*Fusarium solani*	*Agroathelia rolfsii*
Traditional	36.36 ± 24.02(CV = 66.06%)	30.99 ± 26.83 (CV = 86.58%)	15.29 ± 24.56 (CV = 160.63%)	39.91 ± 22.47(CV = 56.30%)
SKIT	54.37 ± 38.53 (CV = 70.87%)	52.84 ± 36.67(CV = 69.39%)	68.65 ± 9.19 (CV = 13.39%)	64.79 ± 5.14 (CV = 7.93%)
Percent improvement of SKIT over Traditional	49.53%	70.50%	349.00%	**62.34%**
p-value (0.05)	NS	<0.05	<0.0001	<0.0001

**Table 8:** Differences between traditional and Serial Knockout Isolation Technique (SKIT).

Description	Traditional	SKIT
Isolation strategy	Serial dilution followed by bacterial isolation in common media like nutrient agar or Luria Bertani agar	Use the Serial Enrichment Isolation Technique (SEIT) followed by bacterial isolation in specific media.
Approach	Non-targeted	Siderophore, chitinase, or cellulase-based pre-filtering, combined with the triple sugar iron test and HCN production assay.
Chances of a human pathogen	More chances of recovery	Reduce the likelihood of recovering Enterobacteriaceae, a group that includes some human pathogens, using the triple sugar iron (TSI) assay. However, 16S rDNA and safety tests are needed before field application.
Isolation of multi-antagonist bacteria	A large number of recovered bacteria need to be tested against each pathogen separately.	This approach prioritizes the most important pathogen, then eliminates or knocks out non-antagonists in the next step.
Plant growth promotion test	May or may not	Since siderophore producers are selected only at the initial step, and siderophore producers often have plant growth-promoting traits, but this requires confirmation.
Time and resources	Minimum 70 days needed and involved labor and lab resources.	SKIT is proposed to reduce workflow time (only 45 days) compared to traditional screening and save resources and labor.
Results	Both non-efficient and efficient multi-antagonist bacteria are on the plate	Most likely, non-human pathogenic potential multi-antagonist bacteria
Limitation	Both volatile and non-volatile mechanisms of controlling phytopathogens were recovered.	Only siderophore producers with or without volatile-producing bacteria would be recovered.
Validation	Validation showed that a high number of unique bacteria were recovered, requiring more time, labor, and lab resources for screening. Based on the TSI assay, recovered isolates were more likely Enterobacteriaceae, with fewer multi-antagonists and lower potential to inhibit phytopathogens.	The SKIT approach allows for high-potential multi-antagonist combinations, fewer bacteria per sample, and is less likely to select for Enterobacteriaceae than traditional approaches.

## Limitations

Siderophore production and the ability to produce hydrolytic enzymes such as chitinase, cellulase, and protease are important traits for controlling phytopathogens. SEIT provides a robust technique for isolating antagonistic bacteria by first isolating siderophore-producing bacteria (SPB) or hydrolytic enzymes (e.g., cellulase, chitinase, or protease), which serve as key traits for screening initial bacteria from the sample. Further, these bacteria may be screened using TSI assays to eliminate human pathogens; the remaining isolates are assessed for the HCN test, and then these bacteria are serially assessed in a dual-assay, knockout manner for antagonistic activity against the predominant pathogen in decreasing order. However, TSI removes Enterobacteriaceae, but many human pathogens are not Enterobacteriaceae, e.g., *Pseudomonas aeruginosa* and *Bacillus cereus*. The only limitation of the SKIT approach is that antagonistic bacteria that use mechanisms other than siderophore or cell wall-degrading enzyme mechanisms, such as controlling phytopathogens through volatile emissions or other means, may not be recovered. To date, no reports on the percentage of antagonistic bacteria that emit volatile compounds or on mechanisms involving siderophore and chitinase in disease suppression have been published, so we were unable to quantify the percentage of antagonists missed by the SKIT approach. However, this strategy may isolate multi-antagonist bacteria more quickly, with less time, labor, and resources than the traditional method. Since the TSI and HCN assays were less costly or less resource-demanding, we first emphasize the elimination of probable human opportunistic pathogens. However, finally screened bacteria should be characterized through 16S rDNA and biological safety tests before their field application.

## Ethics statements

The present manuscript does not involve human or animal subjects or any data collected from social media platforms.

## Related research article

None.

## Declaration of competing interest

Please tick the appropriate statement below (please do not delete either statement) and declare any financial interests/personal relationships which may affect your work in the box below.

The authors declare that they have no known competing financial interests or personal relationships that could have appeared to influence the work reported in this paper.

## Data Availability

Data will be made available on request.

## Appendix Supplementary materials

Image, application 1
