# Supplementary material for: Serial Knockout Isolation Technique (SKIT): targeted isolation of multi-antagonist bacteria
Source: MethodsX. 2026 Jul 31;17:104082. doi: 10.1016/j.mex.2026.104082 (PMC13474688; doi:10.1016/j.mex.2026.104082)
Supplement: Supplementary file 1 [file mmc1.docx]

**Table S1. Phytopathogens' priority list of the different crops.**

| **Sr .no** | **Crop** | **Pathogen** | **Description** | **References** |
| --- | --- | --- | --- | --- |
| 1 | Soybean | *Phakopsora pachyrhizi* > Brown spot > Seedling diseases > anthracnose > Charcoal rot.  *Macrophomina phaseolina* > *Rhizoctonia solani* in central India | Across all eight predominant soybean-producing countries  Central India | [22]  [1] |
| 2 | Mustard | Alternaria blight > Powdery mildew > white rust > Sclerotinia rot | Across all India | [23] |
| 3 | Sunflower | Rust > *Phomopsis* stem canker > Sclerotia head rot > *Rhizopus* head rot > Downy mildew. | Seven midwestern USA states and studies between 2002 and 2015 | [24] |
| **4** | Chickpea | *Fusarium* wilt > Dry root rot > collar rot  *Aschochyta* blight > *Botrytis* gray mold > Stem rot > *Alternaria* blight | Soil-borne diseases  Foliar diseases | [25] |
| 5 | Groundnut | Collar rot > Stem rot > alfaroot | Soil-borne diseases | [26] |
